# The worldwide burden of smoking‐related oral cancer deaths

**DOI:** 10.1002/cre2.265

**Published:** 2019-11-26

**Authors:** Riccardo Nocini, Giuseppe Lippi, Camilla Mattiuzzi

**Affiliations:** ^1^ Section of ENT, Department of Surgical Sciences, Dentistry, Gynecology and Pediatrics University of Verona Verona Italy; ^2^ Section of Clinical Biochemistry, Department of Neuroscience, Biomedicine and Movement University of Verona Verona Italy; ^3^ Service of Clinical Governance Provincial Agency for Social and Sanitary Services Trento Italy

**Keywords:** cancer, mortality, smoking, lip, oral cavity

## Abstract

**Objectives:**

Although it is now established that cigarette smoking enhances the risk of oral malignancies, less is known on this epidemiologic interplay. Therefore, this brief report aims to provide an update on the worldwide burden of smoking‐related deaths for lip and oral cavity cancers.

**Material and methods:**

We performed an electronic search in Global Health Data Exchange registry using the keywords “lip and oral cavity cancer” and “smoking,” combined with “deaths,” “year,” and “location.”

**Results:**

Global mortality for lip and oral cavity cancers has considerably grown during the past three decades, exhibiting a 1.40‐fold increase. Although up to one third (i.e.,30.5%) of worldwide deaths for these malignancies are still attributable to cigarette smoking, smoking‐related mortality for oral malignancies has decreased during the past three decades. The impact of cigarette smoking on these deaths is lower (i.e.,18.7%, gradually decreasing) in Africa, whereby the burden is higher in Europe (i.e.,43.7%) and Western Pacific (40.9%, gradually escalating).

**Conclusions:**

Despite recent policies of smoking dissuasion may have contributed to mitigating the negative impact of smoking on oral cancers, additional healthcare interventions shall be planned to reduce the still high mortality, especially in Western Pacific.

## 1 INTRODUCTION

Several lines of evidence now indicate that tobacco exposure, especially conventional (i.e.,combustible) but also e‐cigarette smoking, enhances the risk of developing many forms of cancers, especially malignancies of lung (Bold et al., 2018), lips and oral cavity (Flach, Maniam, & Manickavasagam, 2019; Nouraei, 2019). Notably, prevention of smoking‐related cancers encompasses a variety of individualized interventions other than nicotine replacement therapy (D'souza & Addepalli, 2018). Although recent analyses have been published on the relationship between cigarette smoking and lung cancer (Bold et al., 2018), less is known on the epidemiology cigarette smoking‐related attributable risk of developing oral malignancies, to the best of our knowledge. Therefore, this brief report aims to provide an update on the worldwide burden of smoking‐related deaths for lip and oral cavity cancers.

## 2 MATERIALS AND METHODS

We performed an electronic search in the Global Health Data Exchange (GHDx) registry, a large database of health‐related data maintained by the Institute for Health Metrics and Evaluation (Institute for Health Metrics and Evaluation, 2019 http://ghdx.healthdata.org/gbd-results-tool), which provides updated statistics on 84behavioral, environmental, occupational, and metabolic risks factors for 354diseased conditions in 195countries, between the years 1990 and 2017 (GBD 2017 Risk Factor Collaborators, 2018). Our search, on the basis of the keywords “lip and oral cavity cancer” (International Classification of Diseases 10[ICD‐10] Codes C00‐C07, C08‐C08.9, and Z85.81‐Z85.810) and the causal condition “smoking,” was combined with the epidemiologic variables “deaths” (i.e.,mortality rate; per 100,000), “year” (the period from the years 1990 and 2017), and “location” (i.e.,the six worldwide regions). According to the Global Burden of Disease (GBD) 2017 Risk Factor Collaborators (GBD 2017 Risk Factor Collaborators, 2018), the impact of smoking on lip and oral cavity cancer deaths has been estimated from data obtained from five contemporary U.S.cohort studies, including 954,029 individuals (532,651 women and 421,378 men) aged 55years or older, who were followed up for 12years (Carter et al., 2015). The findings of these studies have allowed the conclusion that cigarette smoking is linearly associated with an increasing risk of dying for lip and oral cavity cancers, from 3.2‐fold (odds ratio = 3.19; 95% confidence interval [2.16, 4.36]) in people smoking 17.7 packs per year to 3.7‐fold (odds ratio = 3.75; 95% confidence interval [2.33, 5.4]) in those smoking 35.4 packs per year, 4.0‐fold (odds ratio = 4.02; 95% confidence interval [2.38, 6.00]) in those smoking 53.1 packs per year, 5.5‐fold (odds ratio = 5.52; 95% confidence interval [2.57, 9.80]) in those smoking 70.8 packs per year, up to 7.3‐fold (odds ratio = 7.27; 95% confidence interval [2.60, 14.74]) in those smoking 88.5 packs per year compared to nonsmoking people, respectively. For all smoking attributable health outcomes, 5‐year lagged daily smoking prevalence was used as exposure, while current smokers were defined as individuals currently using any form of smoked tobacco product on daily basis. Current smoking prevalence was finally defined using a spatiotemporal Gaussian process regression, as specified in detail elsewhere (GBD 2017 Risk Factor Collaborators, 2018). The output of our electronic search was downloaded in comma‐separated values (CSV) and imported into an Excel file (Microsoft, Redmond, WA, US) for analysis and graphical representation. The study was performed in accordance with the Declaration of Helsinki.

## 3 RESULTS

**Figure 1:**
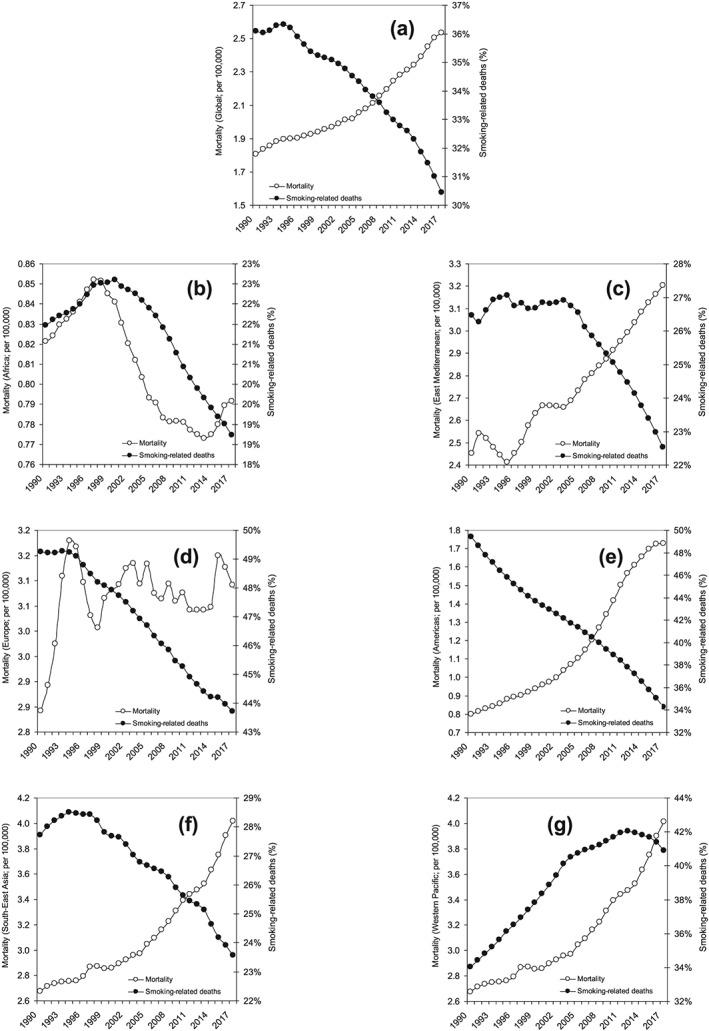
(a)Trends of worldwide incidence of total and smoking‐related lip and oral cavity cancer deaths worldwide, as well as in (b)Africa, (c)East Mediterranean, (d)Europe, (e)Americas, (f) Southeast Asia, and (g)Western Pacific

The results of our search in the GHDx registry are summarized in Figure 1. Overall, the global mortality for lip and oral cavity cancers has considerably grown during the past three decades, exhibiting a 1.40‐fold increase (i.e.,from 1.8 to 2.5 deaths for 100,000). Nevertheless, this notable increment has not been accompanied by a concomitant increase of smoking‐related mortality for these types of cancer, whereby smoking‐related deaths have decreased by approximately 1.16‐fold during the past 30years (i.e.,from 36.1% in 1900 to 30.5% in 2017, respectively). A rather different trend could be observed when the death rate and smoking‐related mortality were analyzed separately across the different worldwide regions, as also shown in Figure 1. The highest number of smoking‐related deaths can be recorded in Europe (43.7%), followed by Western Pacific (40.9%), Americas (34.2%), Southeast Asia (23.6%), Eastern Mediterranean (22.5%), and Africa (18.7%).

The mortality rate has increased in all worldwide regions except Africa, with the highest 30‐year increment recorded in the Western Pacific (Figure 1). On the other hand, the smoking‐related mortality for lip and oral cavity cancers has exhibited a significant decline in all worldwide regions except Western Pacific, where the impact of smoking on these cancer‐related deaths has increased by 1.20‐fold (i.e.,from 34.0% to 40.9%). Basically, the trend of smoking‐related deaths seems to follow a constantly decreasing pattern in Africa, Eastern Mediterranean, Europe, Americas, and Southeast Asia, while a complete opposite trend could be seen in Western Pacific, at least until the year 2012, since when the trend has apparently flattened and has gradually reversed (Figure 1).

## 4 DISCUSSION

Although the impact of smoking‐related mortality for lip and oral cavity cancers has substantially decreased over time and despite the inherent caveats characterizing this type of analyses, the results of our GHDx database search attest that up to one third (i.e.,30.5%) of worldwide deaths for these malignancies may be still attributable to cigarette smoking. A consistent regional heterogeneity could also be observed. More specifically, the impact of cigarette smoking seems considerably lower (i.e.,18.7% and gradually decreasing) in Africa, whereby the burden seems much higher in Europe (i.e.,43.7%) and Western Pacific (40.9% and gradually escalating up to the year 2012). This data are not really surprising, especially those from the Western Pacific, whereby the tobacco use in this region is still epidemic, as revealed by the recent data of the Global Adult Tobacco Survey (Global Adult Tobacco Survey, 2019 http://gatsatlas.org/). On the other hand, the lower risk observed inSoutheast Asia can be attributed to larger usage of other tobaccoproducts, such as smokeless tobacco (mainly chewable; Sreeramareddy, Pradhan, Mir, & Sin, 2014).

In conclusion, although some recent policies of smoking dissuasion have contributed to mitigating the adverse health impacts of smoking in some countries (Frazer et al., 2016), additional healthcare interventions shall be urgently planned to reduce the still high impact of cigarette smoking on the worldwide burden of lip and oral cavity cancers, especially in regions where the burden remains higher (i.e.,Europe and Western Pacific). These would basically include advices from healthcare workers, text messaging, proactive telephone support and helplines, self‐help informative material, along with nicotine replacement therapy (West et al., 2015), and reinforcement of global interventions such as the World Health Organization Framework Convention on Tobacco Control (WHO‐FCTC).

## CONFLICT OF INTEREST

The authors declare that they have no competing interests.
